# Early Response to the Plant Toxin Stenodactylin in Acute Myeloid Leukemia Cells Involves Inflammatory and Apoptotic Signaling

**DOI:** 10.3389/fphar.2020.00630

**Published:** 2020-05-08

**Authors:** Daniele Mercatelli, Massimo Bortolotti, Vibeke Andresen, André Sulen, Letizia Polito, Bjørn Tore Gjertsen, Andrea Bolognesi

**Affiliations:** ^1^Department of Experimental, Diagnostic and Specialty Medicine-DIMES, Alma Mater Studiorum, University of Bologna, Bologna, Italy; ^2^Department of Pharmacy and Biotechnology-FaBiT, Alma Mater Studiorum, University of Bologna, Bologna, Italy; ^3^Centre of Cancer Biomarkers CCBIO, Department of Clinical Science, University of Bergen, Bergen, Norway; ^4^Hematology Section, Department of Internal Medicine, Haukeland University Hospital, Bergen, Norway

**Keywords:** acute myeloid leukemia, apoptosis, plant toxins, ribosome-inactivating protein, stenodactylin, toxic lectins, type 2 ribosome inactivating protein

## Abstract

Stenodactylin, a highly toxic type 2 ribosome-inactivating protein purified from the caudex of *Adenia stenodactyla* Harms, is a potential anticancer drug candidate. Previous studies demonstrated that stenodactylin induces apoptosis and necroptosis in treated cells, involving the production of reactive oxygen species. We analyzed the effect of stenodactylin on Raji and Ramos (Human Burkitt’s lymphoma cells) and MOLM-13 (acute myeloid leukemia cells). Moreover, we focused on the early events in MOLM-13 cells that characterize the cellular response to the toxin by whole-genome microarray analysis of gene expression. Treatment with stenodactylin induced the depurination of 28S rRNA within 4 h and increased the phosphorylation of p38 and JNK. A time-dependent activation of caspase 1, 2, 8, 9, 3/7 was also observed. Genome-wide gene expression microarray analysis revealed early changes in the expression of genes involved in the regulation of cell death, inflammation and stress response. After 4 h, a significant increase of transcript level was detectable for ATF3, BTG2, DUSP1, EGR1, and JUN. Increased upstream JUN signaling was also confirmed at protein level. The early response to stenodactylin treatment involves inflammatory and apoptotic signaling compatible with the activation of multiple cell death pathways. Because of the above described properties toward acute myeloid leukemia cells, stenodactylin may be a promising candidate for the design of new immunoconjugates for experimental cancer treatment.

## Introduction

Ribosome-inactivating proteins (RIPs) are a family of cytotoxic RNA N-glycosylases from plants able to hydrolyze the glycosidic bond of a specific adenine in the eukaryotic ribosomal 28S RNA causing the irreversible translational arrest and consequent cell death (Bolognesi et al., 2016). RIPs are largely distributed in the plant kingdom and many plants producing RIPs have been used for centuries in traditional medicine (Polito et al., 2016a; Bortolotti et al., 2019). RIPs are monomeric or dimeric proteins, classified as type 1 or type 2 depending on the absence or presence of a lectin B-chain, respectively (Bolognesi et al., 2016). Both type 1 and type 2 RIPs possess an A-chain with enzymatic activity. The presence of the B-chain in type 2 RIPs facilitates the binding to the cell surface and mediates the entry of the whole toxin into the cell, making type 2 RIPs more cytotoxic than type 1 (Bolognesi et al., 2016). Besides the well-known activity on 28S rRNA, some RIPs show activity on other nucleotide substrates, such as mRNA, tRNA, DNA, and poly(A). It was then proposed to classify them as polynucleotide:adenosine glycosylases (Barbieri et al., 1997; Battelli et al., 1997; Bolognesi et al., 2002).

RIPs have been widely investigated for their antineoplastic potential and frequently employed as toxic payload to produce a variety of immunotoxins (ITs) after conjugation to a carrier molecule, specifically designed to selectively eliminate a cell population (Polito et al., 2013; Gilabert-Oriol et al., 2014; Akkouh et al., 2015). RIP-containing ITs have been investigated in several pre-clinical models and clinical trials, often achieving promising results, especially in the treatment of hematological neoplasms (Bolognesi et al., 2005; Polito et al., 2011; Polito et al., 2016b; Bortolotti et al., 2018). The encouraging success rate of IT-based therapy in clinical trials, particularly in the leukemia field, has driven efforts toward the investigation of enhanced versions of these anticancer drugs. However, the efficacy of ITs as single agents remains limited, and there is a need to integrate more strategies, as combination therapy, in future studies (Wayne et al., 2014; Weidle et al., 2014; Tyagi et al., 2015; Madhumathi et al., 2016; Giansanti et al., 2018; Valent et al., 2019).

Among type 2 RIPs, the most known being ricin (Polito et al., 2019), stenodactylin is a highly toxic lectin purified from the caudex of *Adenia stenodactyla* Harms (Pelosi et al., 2005; Stirpe et al., 2007). Due to its elevated cytotoxicity, especially toward nervous cells, it is considered to be among the most cytotoxic RIPs discovered so far, and an attractive molecule for the production of ITs (Monti et al., 2007; Polito et al., 2016c). Structurally, stenodactylin consists of two chains linked by a disulfide bond, where the A-chain shows the enzymatic activity toward the 28S rRNA, and the B-chain binds the glycan structures on cell surface (Tosi et al., 2010). The separated A-chain of stenodactylin was shown to retain the ability to inhibit *in vitro* protein synthesis, an important feature that makes this protein an attractive candidate for targeted drug delivery. Stenodactylin has been also shown to possess a high enzymatic activity toward ribosomes and herring sperm DNA (hsDNA) substrates, but not on tRNA nor on poly(A) (Stirpe et al., 2007).

The knowledge of the mechanism of action of the toxic payload allows a better design of ITs to achieve specificity in targeting and more potency in destroying cancer cells. Furthermore, it allows predicting synergistic toxic effects in combination with conventional or experimental targeted therapies to develop more effective combination regimens, or to design the more appropriate carrier for delivery (Bornstein, 2015; Polito et al., 2017). Despite several studies on RIPs cytotoxicity, a complete comprehension of the mechanism underlying induction of cell death is still missing. It has been observed in several *in vitro* and *in vivo* models that RIPs, both type 1 and 2, induce apoptosis in intoxicated cells (Narayanan et al., 2005). In addition to apoptosis, increasing evidences suggest that these plant toxins elicit alternative molecular mechanisms that trigger different cell death programs (Polito et al., 2009; Bora et al., 2010; Pervaiz et al., 2016; Polito et al., 2016c). Besides protein synthesis inhibition, RIPs and other ribotoxins have been shown to activate a MAPK-driven proinflammatory and proapoptotic response, termed the ribotoxic stress response (Iordanov et al., 1997; Jandhyala et al., 2008; Jetzt et al., 2009; Zhou et al., 2014) and inflammasome activation (Lindauer et al., 2010) in different cellular models. In some cases, another stress response has been shown to contribute in different manners to inflammation and proapoptotic signaling during RIP intoxication, i.e. the unfolded protein response (UPR) following endoplasmic reticulum (ER)–stress (Lee et al., 2008; Horrix et al., 2011). It has also been suggested that some RIPs could produce a direct damage to nuclear DNA (Bolognesi et al., 2012). However, all these features seem to be somewhat RIP and cellular-context specific.

We have previously shown that stenodactylin induces apoptosis and necroptosis in a neuroblastoma cell line. It has been reported that the production of intracellular ROS is a critical feature of stenodactylin-induced cell death in neuroblastoma cells (Polito et al., 2016c), similar to what observed for the type 2 RIP abrin in HeLa, 293 T (Shih et al., 2001) and Jurkat cells (Saxena et al., 2014). In this context, the primary aim of this study was to investigate the early response to stenodactylin in hematological cells, focusing on gene expression and signaling changes occurring soon after exposure to the toxin, in order to ameliorate our understanding of molecular mechanisms underlying susceptibility to stenodactylin-induced apoptosis. Since very few analyses on how RIPs globally affect gene expression have been made so far, we investigated stenodactylin-induced early gene expression changes by a whole-genome gene expression profile analysis approach using acute myeloid leukemia cells MOLM-13 as experimental model.

## Materials and Methods

### Cell Lines

Human Burkitt’s lymphoma (Raji and Ramos) and human acute monocytic leukemia (AML) (MOLM-13) cells (American Type Culture Collection - ATCC), free of pathogenic contaminations, were maintained in RPMI 1640 medium (Sigma-Aldrich Co., St Louis, MO, USA) containing 10% heat-inactivated fetal bovine serum (FBS), 2 mM L-glutamine, 100 U/ml penicillin and 100 µg/ml streptomycin (Sigma-Aldrich), hereafter named complete medium. All the cultures were kept under standard incubation conditions (humidified atmosphere, 5% CO_2_, 37°C) and passed from two to three times a week to keep them in logarithmic growth phase.

### Reagents and Antibodies

Stenodactylin was purified from the caudex of *Adenia stenodactyla* Harms as previously described (Stirpe et al., 2007); the purity grade was > 99%. CellTiter 96^®^ Aqueous Non-Radioactive Cell Proliferation Assay was obtained from Promega Corporation (Madison, WI, USA). PhosSTOP - Phosphatase Inhibitor and complete ULTRA protease inhibitor cocktail were purchased from Roche Applied Science (Penzberg, Germany). The primary antibodies against phospho-SAPK/JNK (Thr183/Tyr185; 81E11), p38, phospho-p38 (Thr180/Tyr182; 12F8), p44/42 MAPK (Erk1/2), COX IV, and the secondary horseradish peroxidase-conjugated anti-mouse and anti-rabbit IgG were purchased from Cell Signaling Technology, Inc. (Danvers, MA, USA). The primary antibody against caspase 3 and p-ERK 1/2 were purchased from Santa Cruz Biotechnology, Inc (Santa Cruz, CA, USA). Antibodies were diluted following manufacturer’s instructions. Phospho flow cytometry was performed with Alexa Fluor^®^ 647 conjugate mouse antibodies against phospho-p38 (Thr180/Tyr182; clone 36/p38), phospho-JNK (Thr183/Tyr185; clone N9-66), phospho-ERK1/2 (Thr202/Tyr204; clone 20A), and IgG isotype κ control (clone MOPC-21) purchased from BD transduction Laboratories (Heidelberg, Germany) (Mercatelli, 2015).

### Cell Protein Synthesis Inhibition Assay

The inhibitory activity of stenodactylin was evaluated as inhibition of L-[4,5-^3^H] leucine incorporation. Cells (4 × 10^4^/well) were seeded in 96-well microtiter plates in 100 µl of complete medium in the presence or absence (untreated controls) of 100 µl of stenodactylin added to final concentration ranging from 10^-9^ to 10^-13^ M. At different time points, 1µCi of L-[4,5-^3^H] leucine was added to each well. After further 6 h, cells were harvested with an automatic cell harvester (Skatron Instruments, Lier, Norway) onto glass-fiber diskettes. Cell-incorporated radioactivity was determined by a β-counter with ReadyGel scintillation liquid (Beckman Instrument, Fullerton, USA) containing 0.7% acetic acid. Cell protein synthesis inhibitory rate was expressed as the percentage of untreated controls, and concentrations inhibiting 50% of protein synthesis (IC_50_) of stenodactylin were calculated (Mercatelli, 2015).

### Cell Viability Assay

Cell viability was assessed by 3-(4,5-dimethylthiazol-2-yl)-5-(3-carboxymethoxy-phenyl)-2-(4-sulfophenyl)-2H-tetrazolium, inner salt (MTS) reduction assay, using the CellTiter 96^®^ Aqueous Non-Radioactive Cell Proliferation Assay. Briefly, cells (4 × 10^4^/well) were seeded in 96-well microtiter plates (BD Falcon, Franklin Lakes, NJ, USA) in 100 µl complete medium. Cells were then incubated in the absence (untreated controls) or in the presence of stenodactylin, at the desired concentrations with additional 100 µl of complete medium. After the indicated times, 20 µl/well of MTS were added to each well and incubated at 37°C for 1 h and the absorbance was measured at 492 nm. All assays were performed in triplicate and repeated in at least three independent experiments. Cell survival rate was expressed as the percentage of untreated controls and half-maximal effective concentration (EC_50_) of stenodactylin was calculated by linear regression.

### cDNA Synthesis and qRT-PCR for Apurinic Sites

For detection of apurinic sites in the 28S rRNA, the quantitative Real-Time PCR (qRT-PCR) method described by Melchior and Tolleson (Melchior and Tolleson, 2010) was applied with some modifications. Briefly, 800 ng of total RNA were reverse transcribed with the iScript cDNA Synthesis kit (Bio-Rad) following the manufacturer’s instructions, applying 4 μl of 5× iScript Mix, 1 μl of iScript reverse, the sample and Nuclease free water to a total volume of 20 μl. The reaction mix was incubated for 5 min 25°C, followed by 30 min incubation at 42°C, then by 5 min at 85°C. The cDNA was then stored at −20°C. Three μl of a 1:125 dilution of the resulting cDNA were used for qRT-PCR, which was performed in 20 μl of reaction mixture consisting of 10 μl of 2× EvaGreen Supermix (Bio-Rad), 1 μl of each primer (final concentration of 0.4 μM), 3 μl of template and 6 μl of Nuclease free water. A sequence of the 28S rRNA near to the apurinic site served as internal control. The following primers were used: 28S rRNA control, 5′-GATGTCGGCTCTTCCTATCATTGT-3′ (forward); 28S rRNA control, 5′-CCAGCTCACGTTCCCTATTAGTG-3′ (reverse); 28S rRNA depurination, 5′- TGCCATGGTAATCCTGCTCAGTA-3′ (forward); 28S rRNA depurination, 5′- TCTGAACCTGCGGTTCCACA-3′ (reverse). qRT-PCR was performed using the CFX96 Bio-Rad Real-Time System and the following cycling program: enzyme activation for 30 s at 98°C, 44 cycles of denaturation for 3 s at 98°C and annealing/extension for 8 s at 60°C, and melt curve for 5 s/step at 65°C–95°C (in 0.5°C increments). The relative gene expression changes (given as fold changes compared to untreated controls, which were set to 1) were calculated with BioRad CFX Manager software using the ΔΔCt method. The data represent mean ± SD of three independent experiments, each performed in duplicate (Mercatelli, 2015).

### Cell and Nuclear Morphology

Cells (3 × 10^4^/well) were seeded in 24-well plates in 500 µl of complete medium in the presence or absence (untreated controls) of 500 µl of stenodactylin at final concentration of 10^-9^ M. After 48 h, the morphological analysis was conducted through phase contrast microscopy directly in 24-well plates using a digital camera from Motic Microscopes, (Xiamen, Fujian, China).

The nuclear morphology was evaluated using fluorescence microscopy. Briefly, cells (3 × 10^4^/well) were seeded in 24-well plates in 500 µl of complete medium in the presence or absence (untreated controls) of 500 µl of stenodactylin at final concentration of 10^-9^ M. Subsequently, the cells were collected from each well, centrifuged at 500 × g for 5 min, washed once with PBS and fixed with methanol:acetic acid 3:1 for 30 min at room temperature. After a further wash with PBS, cells were pelleted, loaded onto glass slide and incubated with 7 µl DAPI (4´,6-diamidino-2-phenylindole)/antifade and visualized using a Nikon Eclipse E600W fluorescence microscope (Nikon, Melville, NY, USA) (Mercatelli, 2015).

### Analysis of the Mitochondrial Transmembrane Electrical Potential Gradient

The mitochondrial Δψm was examined after staining MOLM-13 cells with the cationic, lipophilic dye JC-1 contained in the Mitochondria Staining Kit (Sigma-Aldrich), which upon aggregation exhibits a fluorescence emission shift from 530 nm (green monomer) to 590 nm (red “J-aggregates”=healthy cells). The cells (1 × 10^5^/1 ml) were seeded in 6-well plates and treated with 1 ml of stenodactylin 10^-9^ M for 24 h. Control samples were carried out adding 1 ml of complete medium. Subsequently, cells were stained with 500 µl of JC-1 dye (1:100 in RPMI) and incubated at room temperature in the dark for 10 min, as previously described (Polito et al., 2016c). The cells were washed three times and observed under the Nikon Eclipse E600W fluorescence microscope.

### Caspase Activity Assay

The caspase 2, 3/7, 8, and 9 activities were assessed by the luminescent assays Caspase-Glo™ 2 Caspase-Glo™ 3/7, Caspase-Glo™ 8, and Caspase-Glo™ 9 (Promega). Cells (1 × 10^4^/well) were seeded in 96-well white-walled microtiter plates in 50 µl RPMI complete medium containing 10^-9^ M stenodactylin. After incubation for the indicated time, Caspase-Glo™ reagents (50 μl/well) were added and the luminescence was measured by Fluoroskan Ascent FL (Labsystem, Helsinki, Finland) following manufacturer’s instructions (Mercatelli, 2015).

Caspase 1 activation was evaluated using the Caspase 1 Colorimetric Assay Kit (Biovision, Milpitas, CA, USA). Cells (1 × 10^6^/2 ml complete medium) were seeded in 25 cm^2^-flasks and treated with 1 ml stenodactylin at final concentration of 10^-9^ M. After 2, 4, and 6 h incubation, cells were centrifuged at 400 × g for 5 min and resuspended in 50 µl of chilled Cell Lysis Buffer, provided with the kit, and incubated on ice for 10 min. After a centrifugation for 1 min at 10,000 × g, supernatants were transferred to fresh tubes, kept on ice and protein concentration of each sample was determined through Bradford method. Afterwards, each sample was added of 50 µl of Cell Lysis Buffer, 50 µl of 2× Reaction Buffer (containing 10 mM DTT) and 5 µl of the YVAD-pNA caspase 1 substrate (200 µM final concentration). After incubation at 37°C for 2 h, samples were transferred in a 96-well plate and the absorbance at 405 nm was measured using the microtiter plate reader Multiskan EX (Thermo Labsystems).

### Annexin V-Fluorescein Isothiocyanate/Propidium Iodide Staining

The apoptosis/necrosis rate in stenodactylin treated cells was evaluated through flow cytometry analysis. Briefly, cells were stained with Annexin V-fluorescein Isothiocyanate (FITC) and propidium iodide (PI), using the Apoptest™ kit from Nexins Research (Hoeven, the Netherlands) according to the manufacturer’s protocol (Bredholt et al., 2013). Data were collected on a Guava easyCyte™ Flow Cytometer (Merck Millipore, Merck KGaA, Darmstadt, Germany) and analyzed with FlowJo 7.2.5 software (TreeStar Inc., Ashland, OR, USA).

### Intracellular Staining of Proteins and Phospho Flow Cytometric Analyses

MOLM-13 cells (1 × 10^6^) were fixed in 1.6% paraformaldehyde (PFA), permeabilized with 100% methanol and stored at −80°C until flow cytometry analysis. PFA fixed, methanol-permeabilized cells were rehydrated by addition of 2 ml phosphate buffered saline (PBS) pH 7.4, resuspended by vortexing, and then centrifuged. The cell pellet was washed once with 2 ml PBS + 1% bovine serum albumin (BSA) (Sigma-Aldrich), resuspended in 50 μl PBS + 1% BSA, and then split evenly into new cytometry tubes for staining. Then, 50 μl of an antibody mix containing 0.13 μg primary Alexa Fluor^®^ 647 conjugated phospho-specific antibody per sample was added to each tube of cells and staining proceeded for 20 min at room temperature. Stained cells were washed by adding 2 ml PBS + 1% BSA and resuspended in 200 μl PBS. Data were collected on a FACS Fortessa (BD) and analyzed with FlowJo software (Mercatelli, 2015). Median fluorescence intensity (MFI) values were used as basis for data analyses.

### Immunoblotting

The samples for immunoblotting analysis were prepared as follow: cells (3 × 10^6^ MOLM-13 cells) were pelleted, washed twice in 0.9% NaCl and lysed in a lysis buffer containing 5 mM TrisHCl (pH 7.5), 1.5 mM KCl, 2.5 mM MgCl_2_, 1% NP40, 5 mM NaF, 1 mM sodium orthovanadate, cOmplete ULTRA protease inhibitor cocktail, PhosSTOP–Phosphatase Inhibitor Cocktail Tablets (Roche) (50–100 μl lysis buffer per sample). The samples were then transferred into 1.5 ml tubes, kept for 45 min on ice and homogenized by pipetting before centrifugation at 14,000 × g for 20 min. Protein concentration was determined using the Bradford protein assay, following the manufacturer’s instructions (Bio-Rad). The protein samples were added to SDS loading buffer (final: 1% SDS, 10% Glycerol, 12 mM Tris-HCl pH 6.8, 50 mM DTT and 0.1% Bromophenol Blue) and boiled for 10 min. SDS-polyacrylamide gels (4%–20%) were loaded with 30 μg protein per well. After electrophoresis (150 V, 1 h) and electroblotting (100 V, o/n 4°C), the PVDF-membranes (HybondP, Amersham Biosciences, Oslo, Norway) were blocked for 1 h in I-Block Blocking agent (Applied Biosystems, Foster City, CA, USA). Primary antibodies were incubated for 1–2 h at room temperature or overnight at 4°C followed by 1 h washing in TBS-Tween 0.1%. Secondary antibodies conjugated to horseradish peroxidase were diluted in 4%–5% fat-free dry milk in TBS-Tween 0.1% and incubated 1 h at room temperature. After washing for 1 h with TBS-Tween 0.1%, the membranes were developed using Supersignal^®^ West Pico or West Femto Chemiluminiscence Substrate (Pierce Biotechnology Inc, Rockford, IL, USA) according to the manufacturer’s instructions. The membranes were imaged using an ImageQuant LAS 4000 (GE Healthcare), and bands were quantified using ImageLab software (Bio-Rad). Data were exported to Excel spreadsheet, corrected for background and loading control (β-actin or COX IV) intensities.

### Isolation of Total RNA

MOLM-13 cells (4 × 10^6^) were seeded in 75 cm^2^ flasks and then stenodactylin (10^-9^ M) was added to a final volume of 20 ml. After different incubation times, ranging from 2 to 6 h, cells were harvested and collected by centrifugation at 500×g for 5 min at room temperature. Cell pellets were frozen at −80°C, then total RNA was extracted using the RNeasy Plus Minikit (Qiagen, Germany), following manufacturer’s instructions. Amount and quality of the extracted RNA were measured by the NanoDrop^®^ ND-1000 spectrophotometer (NanoDrop Technologies, USA) and the Agilent 2100 Bioanalyzer (Agilent Technologies, USA). For microarray experiments, six biological replicates were collected at each time point (Mercatelli, 2015).

### Microarray Probe Labeling and Illumina Sentrix BeadChip Array Hybridization

Transcriptome analysis was performed using the Illumina iScan, which is based upon fluorescence detection of biotin-labeled cRNA. Using the Illumina TotalPrep RNA Amplification Kit (version 280508, Applied Biosystems/Ambion, USA), 300 ng of total RNA from each sample were reversely transcribed, amplified and Biotin-16-UTP–labeled. The amount (15–52 μg) and quality of labeled cRNA were measured using both NanoDrop spectrophotometer and Agilent 2100 Bioanalyzer. Biotin-labeled cRNA (750 ng) was hybridized to the The Illumina Sentrix BeadChip according to manufacturer’s instructions. The Human HT12 v4 BeadChip targets approximately 47231 annotated RefSeq transcripts.

### Microarray Data Extraction and Analysis

Bead summary data was imported into GenomeStudio to remove control probes and to produce a text file containing the signal and detection p-values per probe for all samples. The text file was imported into J-Express Pro 2012 (http://jexpress.bioinfo.no), and signal intensity values were quantile normalized (Bolstad et al., 2003) and log2 transformed (Mercatelli, 2015). Further bioinformatic analysis was performed using R v3.6.1. The array probes were converted into matched gene symbols according to annotation information. In case of multiple probes corresponding to a single gene, the value of gene expression was designated as the highest value of the probes. After this procedure, 23368 genes were kept in the dataset. Samples were then analyzed using Bayes methods based limma package v3.40.0 (Ritchie et al., 2015), and raw p-values were revised using the Benjamini and Hochberg method to control the false discovery rate. If not stated otherwise, genes showing an absolute log2 Fold Change (log2FC) ≥ 1 and an adjusted p-value < 0.05 were considered as Differentially Expressed Genes (DEGs). Gene Ontology (GO) enrichment analysis was performed using the R package enrichR v2.1 (Kuleshov et al., 2016). Gene Set Enrichment Analysis (GSEA) was performed using fgsea R package v1.10.0. Gene sets were derived from the Broad Institute MSigDB collection (msigdbr package v6.2.1).

### cDNA Synthesis and qRT-PCR

cDNAs were synthesized using the iScript cDNA Synthesis Kit (Bio-Rad) running 500 ng RNA in a total reaction volume of 20 μl. Real Time PCR was performed using primePCR custom assays containing primers of genes of interest (Bio-Rad). Human GAPDH and β-Actin were used as endogenous controls. SsoAdvanced™ Universal SYBR^®^ Green Supermix (Bio-Rad) was run with 3 μl cDNA in 20 μl total reaction volumes. The reaction was performed in a 96-well white plate on a CFX96 Real Time PCR system (Bio-Rad) and the following cycling program: enzyme activation for 30 s at 98°C, 40 cycles of denaturation for 5 s at 95 °C and annealing/extension for 20 s at 60°C, and melt curve for 5 s/step at 65°C–95°C (in 0.5°C increments). All the samples were run in three replicates and the data were analyzed using the ΔΔCt method in CFX Manager software (Bio-Rad). The data represent mean ± SD of two independent experiments, each performed in duplicate. Details of the amplicons were given in S1 (Mercatelli, 2015).

### Statistical Analyses

Statistical analyses were conducted using the XLSTAT-Pro software, version 6.1.9 (Addinsoft 2003). Result are given as means ± SD. Data were analyzed by ANOVA/Bonferroni, followed by comparison with Dunnett’s test (Mercatelli, 2015).

## Results

### Cytotoxicity of Stenodactylin in Raji, Ramos, and MOLM-13 Cells

**Figure 1:**
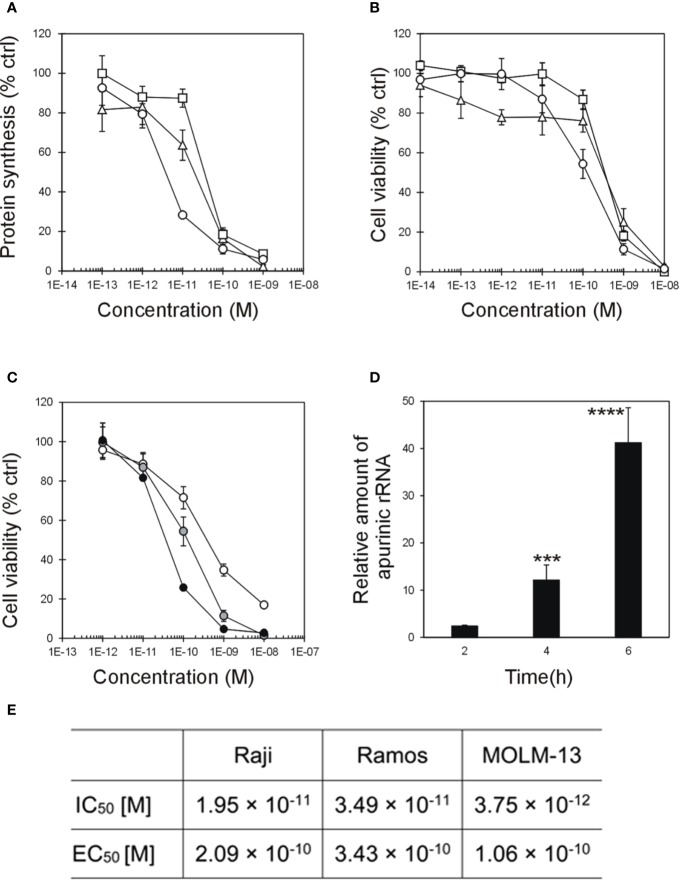
**(A)** Protein synthesis inhibition and **(B)** viability assay on Raji (triangle), Ramos (square), and MOLM-13 (circle) cells treated for 48 h with stenodactylin. **(C)** Viability assay on MOLM-13 cells treated for 24 (white circle), 48 (gray circle, already shown in 1B) and 72 h (black circle) with different concentration of stenodactylin. Results are means of three independent experiments each performed in triplicate. SD never exceeded 10%. **(D)** Depurination of 28S rRNA by 10^-9^ M stenodactylin in MOLM-13 cells. The resulting relative amount of apurinic sites in 28S rRNA, compared to untreated controls, was determined by quantitative Real-Time PCR (qRT-PCR). Data are given as mean fold change ± standard deviation of the mean of three independent experiments, each performed in duplicate. ***p = 0.0003; ****p < 0.0001 (Mercatelli, 2015). **(E)** Table summarizing concentrations inhibiting 50% of protein synthesis (IC_50_) and the half-maximal concentration reducing cell viability (EC_50_).

Protein synthesis inhibition and cytotoxic activity of stenodactylin were investigated in three hematological cell lines: the lymphoblastic Burkitt’s lymphoma derived Raji and Ramos cells and the AML derived MOLM-13 cells. Protein synthesis inhibition assay was performed in the presence of 10^-13^-10^-9^ M stenodactylin. After 48 h of continuous exposure to 10^-9^ M stenodactylin, protein synthesis was almost completely inhibited in all tested cell lines (**Figure 1A**). However, in these experimental conditions, MOLM-13 cells resulted more sensible to stenodactylin than Raji and Ramos cells, with a difference of about one logarithm in the IC_50_ values (**Figures 1A, E**). Cell viability was evaluated by MTS dye reduction and measured after 48 h of exposure to the toxin. In the presence of 10^-14^-10^-8^ M stenodactylin, cell viability decreased in a concentration-dependent manner (**Figure 1B**). Despite a marked difference reported in the efficacy of stenodactylin to inhibit protein synthesis in the three cell lines, viability assays showed that all tested cell lines were similarly sensitive to the toxin, with very close EC_50_ values (**Figure 1E**). Time-course dose-response experiments showed a time and dose dependent reduction in MOLM-13 cell viability (**Figure 1C**) (Mercatelli, 2015).

MOLM-13 was the cellular model chosen to investigate stenodactylin-induced cell death at gene and protein level. The concentration of 10^-9^ M, i.e. the concentration causing a complete inhibition of protein synthesis after 48 h of incubation, was chosen for the analysis of MOLM-13 cells at early time point treatment. A time-dependent increase of apurinic sites in 28S rRNA was detected upon stenodactylin treatment at short times of incubation. A significant increase in the relative amount of apurinic rRNA was observed after 4 h (12.1 ± 3.2-fold) and 6 h (41.2 ± 7.4-fold). No significant difference compared to control was observed after 2 h (**Figure 1D**) (Mercatelli, 2015).

### Stenodactylin-Induced Apoptotic Features in MOLM-13 Cells

**Figure 2:**
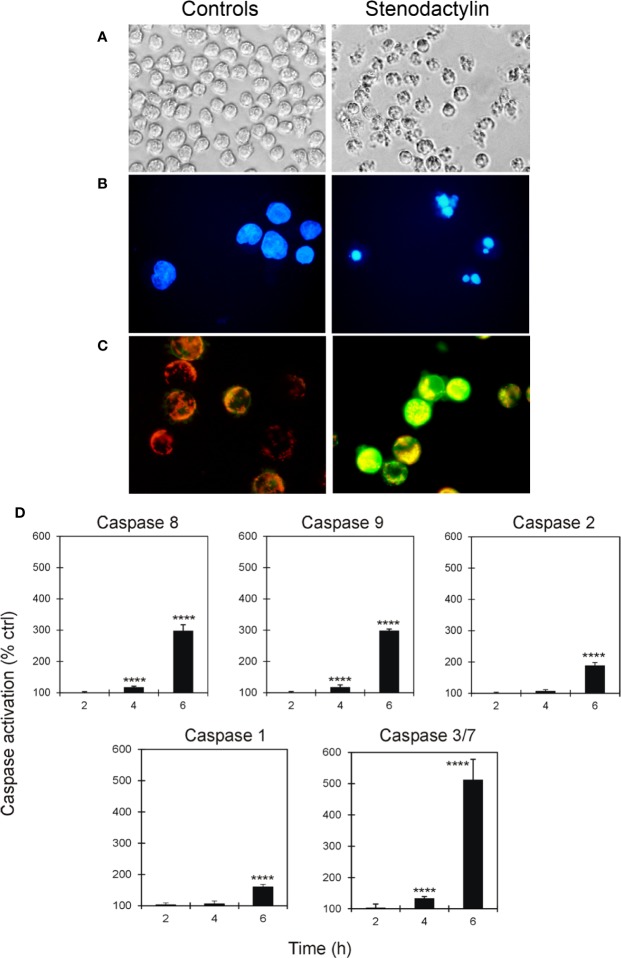
Activation of apoptosis induced by stenodactylin in MOLM-13 cells. Cells were cultured in the absence (controls) or in the presence of 10^−9^ M stenodactylin. Cells morphology was assessed after 48 h of intoxication using phase contrast microscopy (magnification 400×) **(A)** and fluorescence microscopy after incubation with DAPI (magnification 600×) **(B)**. Mitochondrial transmembrane potential of cells was evaluated after 24 h by staining with JC-1 and analysis through fluorescence microscopy (magnification 600×) **(C)**. Caspase activation was evaluated through luminescent (caspase 8, 9, 2 and 3/7) or colorimetric (caspase 1) assays **(D)**. Caspase activity is expressed as percentage of control values. Data are given as mean fold change ± standard deviation of the mean of three independent experiments, each performed in duplicate (Mercatelli, 2015). Data were analyzed by ANOVA/Bonferroni test, followed by Dunnett’s comparison (confidence range 95%; ****p ≤ 0.0001).

The presence of cellular and nuclear morphological changes in MOLM-13 cells treated for 48 h with stenodactylin were evaluated by phase contrast microscopy and fluorescence microscopy. In these cells morphological features compatible with apoptosis were detected (**Figure 2A**), including cell shrinkage, membrane blebbing and cytoplasmic condensation. DAPI staining confirmed the presence of nuclear alteration (**Figure 2B**). Disruption of mitochondrial membrane potential was detected at 24 h by JC-1 staining. JC-1 formed characteristic J-aggregates in intact mitochondria, yielding red fluorescence, while in stenodactylin-treated cells mitochondrial depolarization consequent to membrane permeability transition, led to green fluorescence caused by monomeric JC-1 (**Figure 2C**). Caspase activity of MOLM-13 cells exposed to stenodactylin significantly increased after 4 h for caspase 8, 9, and 3/7, and after 6 h for caspase 1 and 2 (**Figure 2D**).

**Figure 3:**
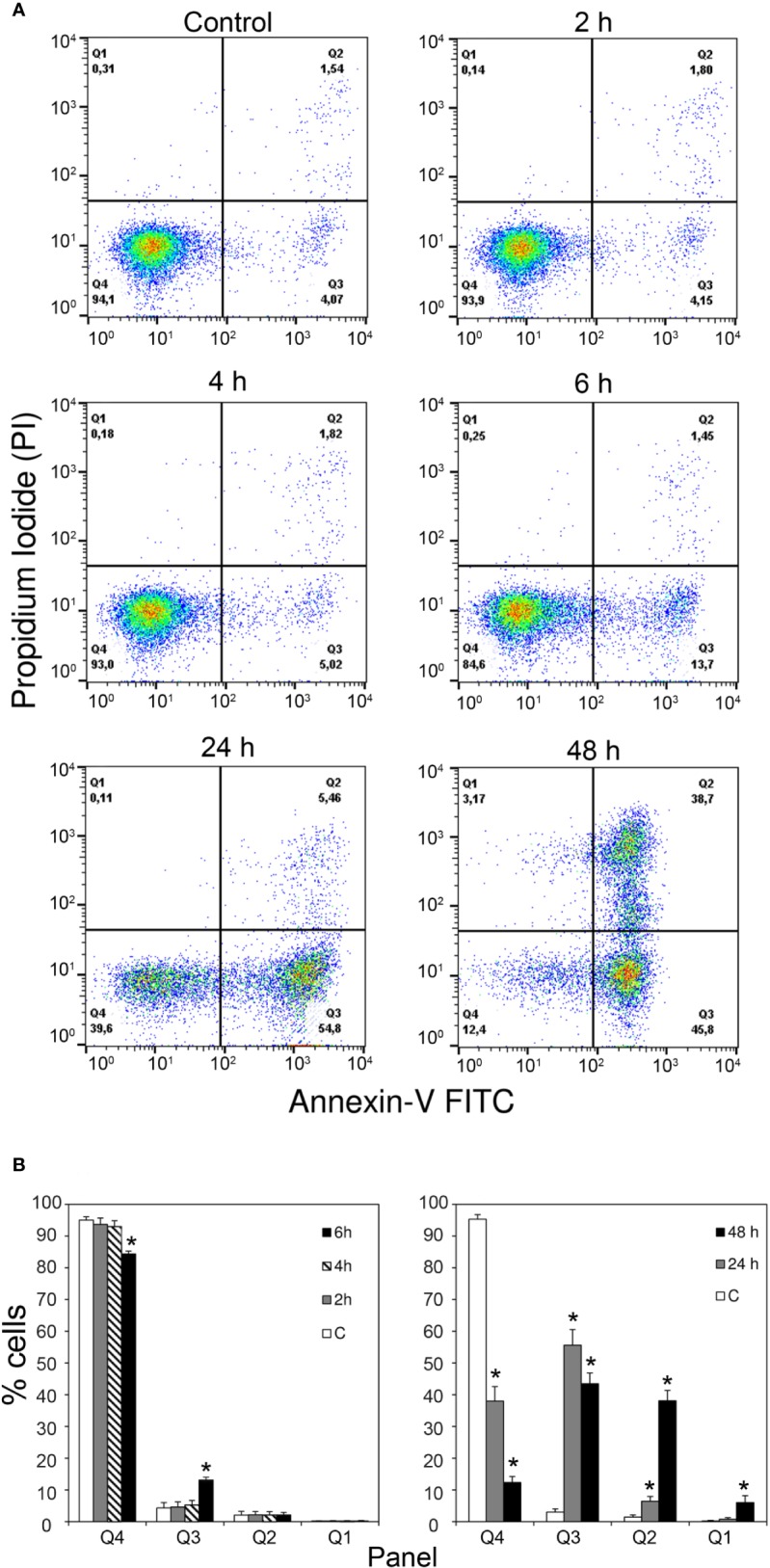
**(A)** Flow cytometric analysis of apoptosis in MOLM-13 cells measured by using Annexin V- propidium iodide (PI) double staining. Representative cytometric dot-plots images of MOLM-13 cells obtained after incubation with stenodactylin (10^-9^M) at different time points. Each specimen presents: viable cells (left down corner, Q4); early apoptotic cells (right down corner, Q3); late apoptotic cells (right upper corner, Q2) and necrotic cells (left upper corner, Q1). **(B)** Quantitative representation of the means of six independent experiments of Annexin V-PI flow cytometric analysis (Mercatelli, 2015). Significance of the difference is indicated by *p < 0.0001 (ANOVA/Bonferroni).

Annexin V-PI double staining, analyzed by flow cytometry, also identified apoptotic changes. Consistently with the time-dependent increase in caspase activity reported above, MOLM-13 cells treated with 10^-9^ M stenodactylin showed a time-dependent increase in Annexin V positive cells (**Figure 3A**). Quantitative analysis showed a significant increase in Annexin V-positive/PI-negative cells (Q3) after 6 h (13.7% ± 0.9%). Consequently, the percentage of viable cells (Q4) significantly decreased after 6 h, reaching values of 38.0% and 12.4%, after 24 and 48 h, respectively (**Figure 3B**).

### Analysis of MAPK Signaling Pathway

**Figure 4:**
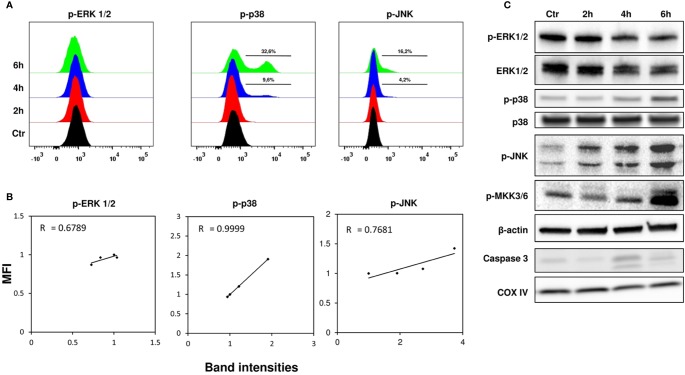
**(A)** Phospho flow analysis of MOLM-13 cells with Alexa-Fluor 647 anti-phospho-ERK 1/2, anti-phospho-p38 and anti-phospho-p-JNK, representative plots out of six independent experiments. Control (black), 2 h (red), 4 h (blue), and 6 h (green) samples are represented. **(B)** Pearson correlation between phospho flow MFI (median fluorescence intensity) and band intensities for phospho-ERK 1/2, phospho-p38 and phospho-JNK. **(C)** Western blot analysis of kinases. Cell lysates (40 μg total protein) were separated by SDS-PAGE and immunoblotted. Figure is representative of three independent experiments (Mercatelli, 2015).

The main MAPKs involved in cell signaling, such as ERK, p38, and JNK, were investigated in the early stages of stenodactylin intoxication. Flow cytometry was used to obtain a single-cell profiling of signal transduction using specific antibodies recognizing phosphorylated proteins; western blot analysis was used to further confirm observed changes. Phospho flow analysis (**Figure 4A**) of phosphorylation of ERK1/2 (Thr202/Tyr204) showed no significant differences between stenodactylin-treated and control samples, while western blot analysis (**Figure 4C**) revealed a slight decrease in ERK1/2 phosphorylation, even if a decrease in total ERK 1/2 was also shown. A time-dependent increase in p38 phosphorylation (Thr180/Tyr182) was clearly detectable in both phospho flow and western blot analysis. Percentage of phospho-p38 cells increased significantly after 4 h of exposure to the toxin. Pearson correlation between phospho flow median fluorescence intensity (MFI) and band intensities for phospho-p38 showed a very strong positive correlation (R=0.999) (**Figure 4B**). A time-dependent increase in phosphorylation of JNK (Thr183/Tyr185) was also observed and the same tendency was detected by western blot, even if with different intensity (Mercatelli, 2015). A clear increase in JNK phosphorylation was evident after 2 h by western blot. A time-dependent increase in phospho-MKK3/6 signal and caspase 3 cleavage (19 and 17 kDa fragments) were detected in treated samples (**Figure 4C**).

### Gene Expression Analysis by Microarray

**Figure 5:**
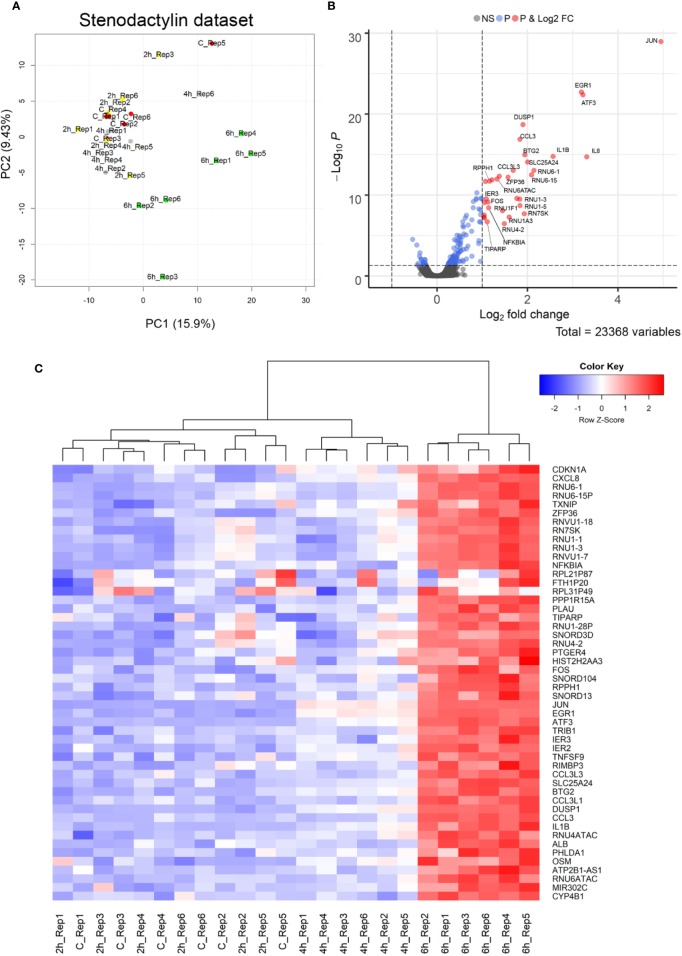
**(A)** Principal Component Analysis (PCA) projection (PC2 vs. PC1) of samples. **(B)** The volcano plot of Differentially Expressed Genes (DEGs). Horizontal dotted line placed at p-value = 0.05 level; vertical dotted lines placed at log2FC = −1 and 1, respectively. Blue dots are genes showing an adjusted p-value < 0.05, while red dots are genes showing both an adjusted p-value < 0.05 and absolute log2FC > 1. **(C)** Heat map of the top 50 genes with an adjusted p-value < 0.05. Samples showing a similar expression pattern are clustered together. The mean normalized expression value of each gene is mapped to a color-intensity value, as indicated by the color bar above.

In order to identify the early effects of stenodactylin on cellular processes, changes in the gene expression profile of MOLM-13 cells treated with stenodactylin 10^-9^ M were analyzed by microarray in a time-course experiment, considering 2-h intervals between 0 and 6 h. The data normalization process and significance cut-offs to identify differentially expressed genes (DEGs) are described in methods. The association between all samples was analyzed by Principal Component Analysis (PCA) (Ringnér, 2008). The first two components of the PCA plot showed a gradient-like placement of samples according to the time of exposure to the toxin, with 2 and 4 h treated samples and controls at one side and 6 h treated samples at the other side (**Figure 5A**).

Considering a significance threshold for adjusted p-value < 0.05, few differentially expressed genes were found to be significantly upregulated after 4 h of exposure to the toxin (JUN, EGR1, ATF3, DUSP1, BTG2). After 6 h, the genes significantly differentially expressed were 164, 144 of them were upregulated and 20 were downregulated. However, only 33 genes having an absolute log2FC > 1 were found to be significantly upregulated (**Figure 5B**). Top 50 significant genes are showed in **Figure 5C**. The complete results of this analysis are given in **Supplementary Table 1**.

**Figure 6:**
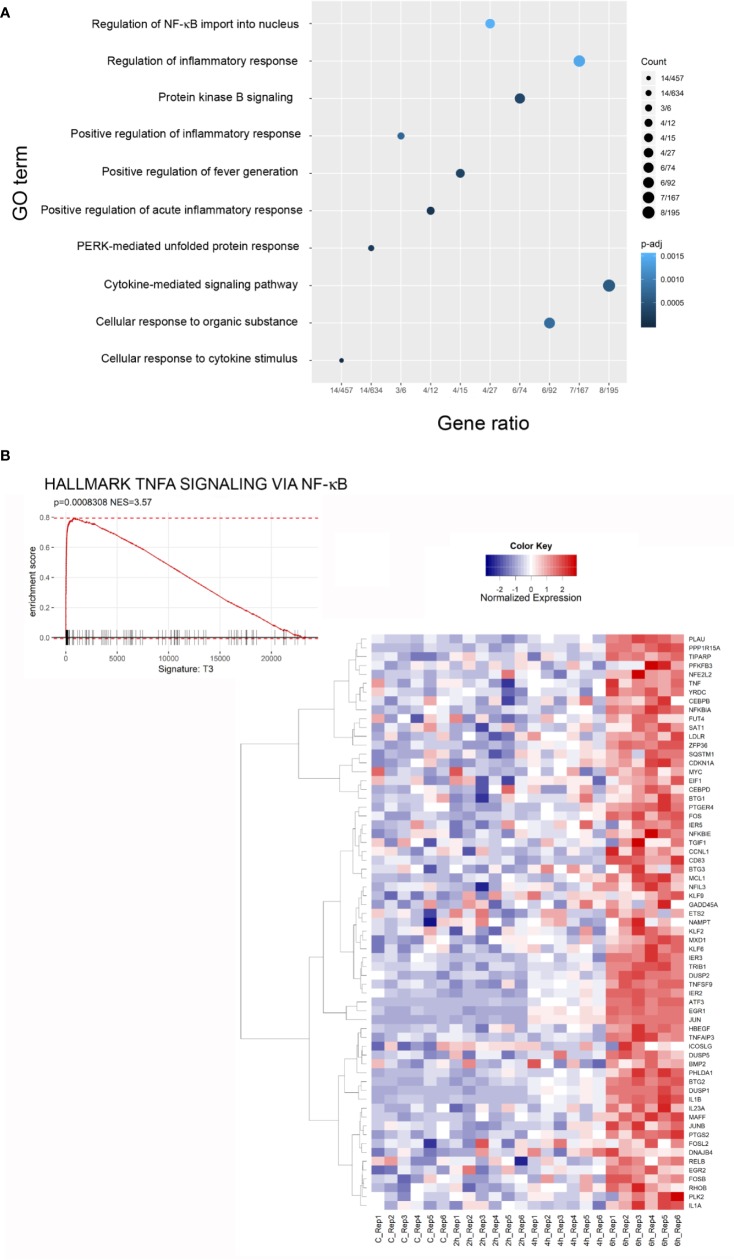
**(A)** Dot plot of the top 10 terms of Gene Ontology (GO) categories of biological process (BP), an adjusted p-value < 0.05 was considered statistically significant. **(B)** Gene Set Enrichment Analysis (GSEA) enrichment plots of the HALLMARK TNFA SIGNALING VIA NFKB from the Broad Institute’s MSigDB in cells treated with stenodactylin for 6 h. The heat map shows the clustered genes in the leading edge of the 6 h subset compared to other time points. The mean normalized expression value of each gene is mapped to a color-intensity value, as indicated by the color bar above. Color intensity is representative of the limma differential expression statistic values vs. control samples (scaled).

**Figure 7:**
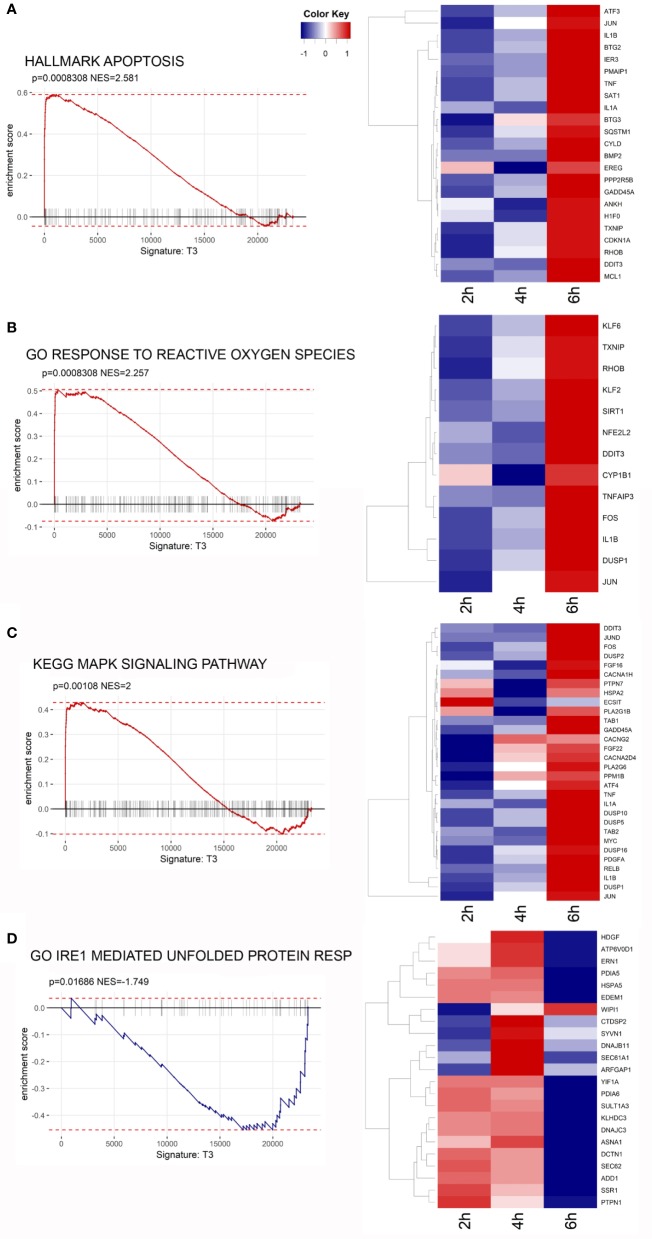
**(A–D)** Gene Set Enrichment Analysis (GSEA) enrichment plots of the indicated gene sets from the Broad Institute’s MSigDB in cells treated with stenodactylin for 6 h. The heat maps show the clustered genes in the leading edge of the 6 h subset compared to other time points. Color intensity is representative of the limma differential expression statistic values vs. control samples (scaled).

The Gene Ontology (GO) Biological Process (BP) category was significantly enriched in GO Terms related to inflammatory processes and stress responses. The top 10 significant GO Terms are showed in **Figure 6A**. Results of the GO analysis are given in **Supplementary Table 2**. To gain more insights into the biological significance of our results, we applied Gene Set Enrichment Analysis (GSEA) to evaluate our microarray data. GSEA confirmed that, after 6 h of exposure to the toxin, most significantly enriched gene sets are related to inflammation, ROS and stress responses. The complete results of GSEA are given in **Supplementary Table 3**. A strong positive enrichment was found with the HALLMARK TNFA SIGNALING VIA NFKB gene set, suggesting the involvement of NF-κB as a mediator of stenodactylin-induced inflammation. In particular, a time dependent increase in gene expression can be observed for ATF3, BTG2, DUSP1, EGR1, EIF1, IER1, IER2, IL1B, JUN (heat map in **Figure 6B**). A strong positive enrichment was found with the HALLMARK APOPTOSIS gene set, confirming the involvement of programmed cell death at transcriptional level (**Figure 7A**). Furthermore, a positive enrichment was found for the GO RESPONSE TO REACTIVE OXYGEN SPECIES gene set, reinforcing the idea that the production of ROS may represent an important feature of stenodactylin induced cell death, occurring soon after exposition to the toxin (**Figure 7B**). A positive enrichment was found with the KEGG MAPK SIGNALING PATHWAY gene set, confirming the involvement of the MAPK cascade in the early response to 28S rRNA damage (**Figure 7C**). Interestingly, a negative association was found with the GO IRE1 MEDIATED UNFOLDED PROTEIN RESPONSE gene set, suggesting that stenodactylin exposure may downregulate some key factors linked to UPR response (**Figure 7D**).

**Figure 8:**
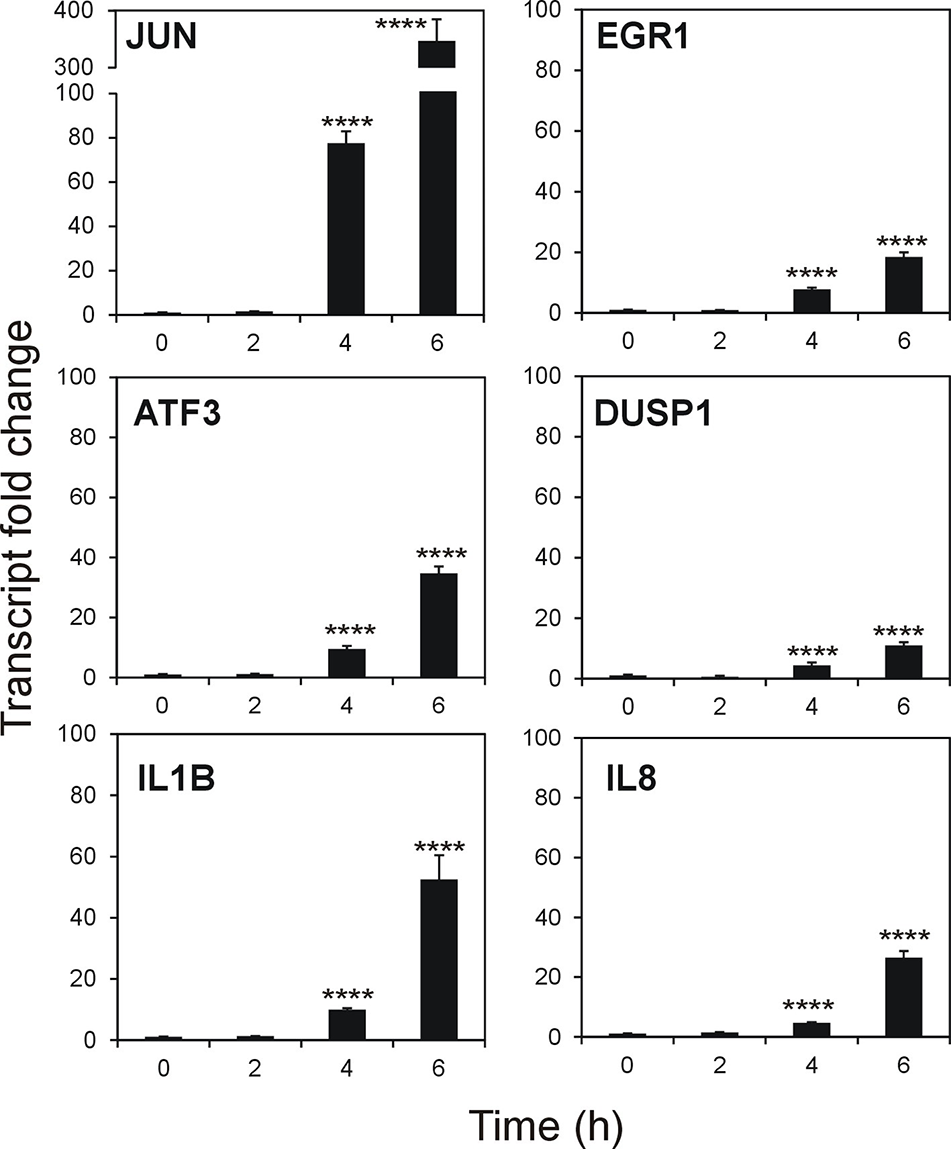
Quantitative Real-Time PCR (qRT-PCR) validation of selected genes from microarray analysis. Data are given as mean fold change ± standard deviation of the mean of two independent experiments, each performed in triplicate (Mercatelli, 2015). Data were analyzed by ANOVA/Bonferroni test, followed by Dunnett’s comparison (confidence range 95%; ****p ≤ 0.0001).

To validate the microarray results on a quantitative level, we studied six of the top DEGs. Time-dependent effect of stenodactylin treatment on the relative expression of JUN, EGR1, ATF3, DUSP1, IL1B and IL8 was assayed (**Figure 8**). Compared to control, the most prominent change in expression was detected for transcription factor JUN, showing a time-dependent increase in expression that reached about 350-fold of the control value. A similar tendency with different intensities was observed for ATF3, EGR1 IL1B, IL8, and DUSP1. Significance of the alteration of expression was proved for all these genes at transcript level (p < 0.0001). According to the microarray analysis, no gene expression for the selected genes was found to be significantly altered after 2 h of treatment with the toxin.

## Discussion

Since its first characterization (Pelosi et al., 2005), stenodactylin has been shown to be among the most toxic RIPs described so far, but the mechanism of cell death induced by this toxin is still not completely understood. Very low concentrations—in the picomolar range—of stenodactylin were previously reported to induce apoptosis and necroptosis in neuroblastoma cells, involving the production of ROS in intoxicated cells (Polito et al., 2016c). Several studies have indicated that different cell death pathways may be involved in RIP-induced cytotoxicity. However, very few reports have investigated the early response to RIP-induced ribosomal damage (Korcheva et al., 2005; Polito et al., 2009; Bora et al., 2010; Leyva-Illades et al., 2010; Horrix et al., 2011; Daniels-Wells et al., 2013; Pervaiz et al., 2016). The present study was designed to evaluate the early response to stenodactylin in a cellular model of blood neoplasia. After evaluation of stenodactylin cytotoxicity in three different human-derived hematological cell lines (Raji, Ramos and MOLM-13), the AML derived MOLM-13 cells were chosen to study gene expression through whole-genome microarray analysis. All the experiments were conducted using stenodactylin at 10^-9^ M, a dose which causes the complete inhibition of protein synthesis after 48 h of incubation. A 12-fold increase in 28S rRNA apurinic sites was detectable after 4 h in stenodactylin–exposed MOLM-13 cells, suggesting that the minimum time required for stenodactylin endocytosis and for its catalytic activity on rRNA is between 2 to 4 h of exposure, consistently with data previously reported for HeLa cells (Battelli et al., 2010). Concurrently, an increase in initiator caspases 8 and 9 and effector caspases 3/7 was detectable after 4 h, while caspase 1 and 2 activities were shown to be significantly higher at 6 h in treated cells, when also apoptotic membrane changes became detectable. It appears that both the intrinsic and the extrinsic pathway are involved in stenodactylin-induced cell death, reinforcing the idea that RIPs induce a complex response in intoxicated cells triggering multiple death pathways (Polito et al., 2009; Pervaiz et al., 2016). Furthermore, it has been recently reported that abrin, another type 2 RIP, can trigger cell death through many mechanisms in different cell lines (Tiwari and Karande, 2019). Stenodactylin treatment induces the disruption of mitochondrial membrane potential, as confirmed by JC-1 positive staining at 24 h. RIP-triggered mitochondria-associated apoptotic signaling was also previously described (Narayanan et al., 2005; Sikriwal et al., 2008; Bora et al., 2010; Polito et al., 2016c).

Stenodactylin treatment significantly altered the MOLM-13 transcriptome affecting cellular stress response pathways related to cell death and inflammation. From GSEA, it seems likely that stenodactylin apoptotic signaling may involve the TNF-α/NF-κB axis. Pro-inflammatory cytokines expression was also enhanced, especially IL1B, IL8, IL1A, and IL23A, suggesting the involvement of pyroptosis following toxin treatment (Gaidt et al., 2016). A similar response involving inflammasome activation was previously reported in ricin-treated macrophages (Lindauer et al., 2010) and in leukemia cells treated with Shiga toxins (Lee at al., 2015). In our study, a mild but significant augment of caspase 1 activity was detectable after 6 h, while effector caspases activity was already at 4 h, thus suggesting a secondary role of caspase-1-dependent pyroptosis in driving cellular responses leading to cell death. As we previously showed in neuroblastoma cells (Polito et al., 2016c), the production of ROS is a key feature of stenodactylin-induced cell death. In the present work, we observed upregulation of genes involved in the cellular response to ROS production, suggesting that oxidative stress may be an early feature of stenodactylin cellular activity. Induction of UPR was reported for some RIPs (Lee et al., 2008; Horrix et al., 2011). Instead, here we showed that stenodactylin downregulates some of the genes involved in the IRE1-mediated UPR. Similarly, ricin toxicity to mammalian cells was previously shown to be enhanced by UPR inhibition (Wang et al., 2011; Pierce et al., 2019).

Five DEGs were identified at 4 h: JUN, EGR1, ATF3, DUSP1, and BTG2. These genes are known to be immediate-early genes, whose activation and transcription usually occur within few minutes after stimulation by extracellular or intracellular signals (Bahrami and Drabløs, 2016). Immediate-early genes have been previously shown to be induced in macrophagic/monocytic cells by ricin (Korcheva et al., 2005), shiga toxin (Leyva-Illades et al., 2010) and other ribosome-targeting toxins, like trichothecene mycotoxin deoxynivalenol in six different human cell lines (Casteel et al., 2010) and pyrrolidine antibiotic anisomycin in HeLa cells (Lu et al., 2006). JUN, ATF3, and EGR1 are well-characterized transcription factors belonging to the AP-1 family, which plays a central role in cell proliferation and transformation and can regulate cytokine and chemokine expression. These transcription factors are often induced in the same cluster by activation of MAPKs due to phosphorylation, which is one of the main up-stream events triggering immediate-early genes. It has been well documented that as a direct consequence of 28S rRNA damage, RIPs may activate the so-called ribotoxic stress response, that is an early event anticipating protein synthesis inhibition, being the latter a late event as it was previously shown in neuroblastoma cells treated with stenodactylin (Polito et al., 2016c). The activation of MAPKs was shown to be involved in ribotoxic stress signaling in immune cells exposed to different protein synthesis inhibitors, thus leading to a proinflammatory response (Tesh, 2012; Jandhyala et al., 2015; Lee et al., 2015). Depending on cell type, exposure to ribotoxins resulted in the alteration of JNK, p38, and ERK signaling (Iordanov et al., 1997; Korcheva et al., 2005; Zhou et al., 2014; Wang et al., 2016). In the present study, we showed that stenodactylin exposure induces a time-dependent increase of p38, JNK 1/2, and MKK 3/6 phosphorylation, while no significant influence was detected on ERK 1/2 activation. Prolonged activation of JNK and p38 MAPKs was previously reported in human myelogenous leukemia cells treated with Shiga toxin type 1, while modest and transient activation of ERK 1/2 was reported (Leyva-Illades et al., 2010). Interestingly, we found that dual specificity phosphatase 1 (DUSP1) was among the first genes induced by stenodactylin. DUSP1 is a negative regulator of MAPKs activity, which dephosphorylates and inactivates MAPK in mammalian cells. Ribotoxic stress response can thus represent a complex reaction to ribosome damage, eliciting a multiple stress-response pathway, which lead to cell death if cellular recovery after ribotoxic insult is not possible.

Different lectins with β-trefoil folding were reported to have antitumor effects both in cell lines (Bovi et al., 2013) and in a melanoma xenografted zebrafish model (Valenti et al., 2020) and to induce apoptosis with the activation of MAPK and stress-activate kinase pathways in addition to NF-kB (Hasan et al., 2019). Moreover, it was previously shown that TNF-α-mediated signaling pathway and apoptosis can be elicited in hematological cells by several lectins upon binding to surface receptors, like globotriaosylceramide (Gb3) carbohydrate chain (Hosono et al., 2014; Liao et al., 2016; Chernikov et al., 2017), which is also a known receptor for Shiga toxins (Lee et al., 2015). Stenodactylin may possess a similar cell entry mechanism, and its B-chain may contribute to the induction of the observed inflammatory response, however we consider most of the stress responses showed in this study to rely on stenodactylin enzymatic activity. In fact, it was previously shown that Shiga toxin enzymatic activity was required to induce p38 and JNK activation in the intestinal epithelial cell line HCT-8, and that inhibition of MAPKs activation prevented cell death (Smith et al., 2003). Furthermore, the sole ricin A chain was found to be fully able to induce apoptosis in MAC-T cells through JNK and p38 signaling pathways (Jetzt et al., 2009). Stirpe and coworkers showed that stenodactylin A chain alone fully retained its enzymatic activity on a cell-free system (Stirpe et al., 2007), but further studies are required to characterize its cytotoxic properties both alone and in conjugation with specific carriers. To date, novel technologies and bioinformatic analysis have fueled an increase in precision oncology approaches, pushing toward a more comprehensive profiling of single patient disease (Gullaksen et al., 2017). Due to its early and powerful cytotoxic effect, stenodactylin represents an attractive tool in cancer therapy both as native molecule and as toxic part of ITs. Due to its high systemic toxicity, native stenodactylin could be used only for loco-regional treatments. Stenodactylin A-chain, linked by chemical conjugation or by genetic engineering, to monoclonal antibodies or antibody fragments specifically designed to target tumor cells, could be used for systemic therapy. Considering the growing interest in personalized medicine, the identification of signaling pathways induced by ribosome damage could help to design tailored drug combinations to enhance cytotoxic potential of RIP-based ITs.

## Conclusions

Stenodactylin elicits a rapid stress response with production of proinflammatory factors and ROS in AML cells leading mainly to apoptosis, but also triggering other cell death pathways. For its elevated cytotoxic potential, stenodactylin may be an ideal candidate to produce immunoconjugates for the experimental treatment of AML and other hematological malignancies. The knowledge of the complex stress response induced by this toxin may help to design new therapeutic combination regimens to enhance the efficacy of currently used anti-AML drugs.

## Data Availability Statement

The raw data supporting the conclusions of this manuscript have been made publicly available by the Authors at NCBI Gene Expression Omnibus (GEO), accession identifier: GSE139401.

## Author Contributions

DM, MB, LP, BG, and AB conceived and designed the experiments. DM, MB, VA, and AS performed the experiments. DM carried bioinformatic analysis. All the authors analyzed the data. DM, MB, and LP drafted the paper. All the authors revised and made the final approval of the version to be submitted.

## Funding

This work was supported by funds for selected research topics from the Alma Mater Studiorum—University of Bologna and by the Pallotti Legacies for Cancer Research; Fondazione CARISBO, Project 2019.0539; DM was supported by Marco Polo Scholarship; BG, AS, and VA were supported by the Norwegian Cancer Society; Solveig & Ole Lunds Legacy.

## Conflict of Interest

The authors declare that the research was conducted in the absence of any commercial or financial relationships that could be construed as a potential conflict of interest.

## Supplementary Material

Supplementary Table 1 Differential Expression Analysis.

Supplementary Table 2 GO Analysis.

Supplementary Table 3 Gene Set Enrichment Analysis.
